# Efficacy of Synthetic Peptide Corresponding to the ACTH-Like Sequence of Human Immunoglobulin G1 in Experimental Autoimmune Encephalomyelitis

**DOI:** 10.3389/fphar.2018.00113

**Published:** 2018-02-23

**Authors:** Valery I. Turobov, Alexey V. Danilkovich, Alexei B. Shevelev, Yulia K. Biryukova, Natalia V. Pozdniakova, Viatcheslav N. Azev, Arkady N. Murashev, Valery M. Lipkin, Igor P. Udovichenko

**Affiliations:** ^1^Branch of Shemyakin and Ovchinnikov Institute of Bioorganic Chemistry, Russian Academy of Sciences, Moscow, Russia; ^2^Emanuel Institute of Biochemical Physics, Russian Academy of Sciences, Moscow, Russia; ^3^N. N. Blokhin Cancer Research Center, Moscow, Russia; ^4^Pushchino Research Center, Russian Academy of Sciences, Pushchino, Russia

**Keywords:** multiple sclerosis, peptide drugs, immunosuppressors, immunocortin, animal model

## Abstract

Peptide immunocortin sequence corresponds to the amino acid residues 11–20 of the variable part of human immunoglobulin G1 (IgG1) heavy chain. Since immunocortin was shown previously to inhibit phagocytosis in peritoneal macrophages and ConA-induced T-lymphocytes proliferation in culture, we suggested that immunocortin administering may be of use for patients with self-immune syndrome. Immunocortin in concentration 10 μM inhibited proliferation of both antigen (myelin)-induced and ConA-induced LN lymphocytes isolated from the lymph nodes of Dark Agouti (DA) rats immunized with chorda shear. The biological trials of the synthetic immunocortin were carried out on the DA rats with induced experimental autoimmune encephalomyelitis (EAE), an animal model of multiple sclerosis. These *in vivo* experiments have shown that intraperitoneal injections of immunocortin in a daily dosage 100 μg per animal reduced symptoms of EAE in DA rats.

## Introduction

Multiple sclerosis (MS) is debilitating autoimmune disease, which affects more than 2 million people worldwide. Typically, the disease begins as a relapsing disorder (relapsing remitting form of MS, RR-MS), which later become a secondary progressive (secondary progressive MS, SP-MS). Inflammatory and neurodegenerative mechanisms play important role in pathogenesis of the onset, and the disease progression; however the molecular mechanisms of the disease pathogenesis remain mostly unknown (Wingerchuk and Weinshenker, 2016). Most of the current approaches for therapy of MS are based on non-specific inhibiting of immunity with immunosuppressive drugs, such as natalizumab, fingolimod, mitoxantrone, and dimethyl fumarate (Mirsky et al., 2016). Additionally, a number of novel therapeutics were developed for MS treatment e.g., ocrelizumab and daclizumab, which affect immune system activity. For example, ocrelizumab, that is formed of humanized monoclonal antibodies (mAb) developed against CD20 marker expressed on outer membrane of the mature B cells, can inhibit proliferation of primed B lymphocytes (Montalban et al., 2016). Another drug, natalizumab, consisted of humanized monoclonal antibodies developed against the protein integrin α4, were shown to inhibit lymphocyte migration (Polman et al., 2006). Daclizumab, as a humanized monoclonal antibodies with a specificity against α subunit of IL-2 receptor (IL2R), can inhibit proliferative activity of primed T cells (Bielekova, 2013). Fingolimod is a non-selective sphingosine-1-phosphate (S1P) receptor modulator FTY720 (Kappos et al., 2010), that is capable of decreasing leucocyte infiltration into the central nervous system (CNS). Mitoxantrone, a cytostatic agent, inhibits proliferation of T, and B lymphocytes (Hartung et al., 2002). Glatiramer acetate (Copaxone) is the only etiotropic anti-MS drug however its clinical efficiency is disputable. Glatiramer acetate is suggested to provide specific blocking anti-myelin antibodies activity, and thus address MS pathogenesis, which is associated with breach of the immune tolerance to myelin (Morris-Downes et al., 2002). Despite the ambiguous data regarding the limited therapeutic efficacy of this drug (Kulakova et al., 2012), glatiramer acetate is widely used for MS therapy. It should be noted that these immune modifying drugs are known for particular low efficacy for treatment of primary, and secondary non-remitting (progressive) forms of MS which are less prevalent than relapsing-remitting form of MS but often associated with more severe clinical symptoms (Gajofatto et al., 2016). Treatment of patients with intravenous immunoglobulins (IVIG) is yet another, well established approach to cure the immune mediated demyelinating neuropathies, including MS (Olyaeemanesh et al., 2016). The therapeutic effect of IVIG is believed to promote specific activation of monocytes and macrophages due to overload of Ig pool in circulation (Bleeker et al., 2001). This activation leads to a forced removing of overall endogenous IgG, including myelin-specific ones. The role of immune system in MS dictates the need to develop new drugs that can inhibit the proliferation of specific lymphocytes.

Experimental autoimmune encephalomyelitis (EAE) is one of the best studied experimental animal models of MS (Steinman, 1996). It is a useful *in vivo* system for the therapeutic management of the disease characteristics by monitoring particular tissue damage mediated by the autoimmune T-cells.

Endogenous peptides, including the products of immunoglobulins proteolysis, appear to be suitable candidates for immune disorder therapeutics (Navolotskaya, 2014). Some of these peptides represent parts of Fc fragment of IgG [tuftsin, residues 289–292 of γ-chain, rigin (341–344), immunorphin (364–373), peptide p2 (335–358)], and are known to promote the immune system activity. In contrast, the immunocortin (adrenocorticotropin, ACTH-like peptide) being also a part of Fab-fragment (11–20) of human Ig γ- or μ-chains, possesses anti-proliferative activity tested on several cell lines (Mitin et al., 1993).

Here we report the effects of synthetic immunocortin on EAE, a model for MS in DA rats. We report that immunocortin is able to suppress the myelin-dependent proliferation of lymphocytes from lymph nodes of EAE animals.

## Materials and methods

### Animals

DA rats (DA/ZFV Crl BR breeding stock was purchased from Charles River Co., Sulzfeld, Germany), were bred at the Branch of Shemyakin and Ovchinnikov Institute of Bioorganic Chemistry, Pushchino, Russia. Animals were kept at the animal facility under the climate-controlled conditions with 12 h light/dark cycles and fed with food, and water provided *ad libitum*.

The study was carried out in accordance with the Institutional Animal Care rules, and User Program, Federal Guidelines SP 2.2.1.3218-14 (Russian Federal Service for Surveillance on Consumer Rights Protection, and Human Wellbeing, 2014); the Guide for the Care, and Use of Laboratory Animals: Eight Edition (National Research Council, 2011), Guidelines for the Care, and Use of Mammals in Neuroscience, and Behavioral Research (National Research Council, 2003), the Directive 2010/63/EU of the European Parliament, and of the Council of 22 September 2010 on the protection of animals used for scientific purposes. The protocol No 521/16 was approved by the Institutional Committee for Ethics on Animal Care, and Use at the Branch of Shemyakin, and Ovchinnikov Institute of Bioorganic Chemistry, Russian Academy of Sciences (Pushchino, 142290, Russia).

Experiments with animals were carried out at GLP certified core facility, the Branch of Shemyakin, and Ovchinnikov Institute of Bioorganic Chemistry at Pushchino, supported by the Russian Ministry of Education, and Science (RFMEFI62117X0018).

### Peptide synthesis

The immunocortin (*H-ValLysLysProGlySerSerValLysVal-OH*), and myelin basic protein MBP p63–81 peptide (*H-AlaArgThrThrHisTyrGlySerLeuProGlnLysSerGlnArgSerGln-OH*) were synthesized using t-Boc protected amino acids as previously described (Mitin et al., 1993). The protected amino acids were consistently synthesized using N,N′-diisopropylcarbodiimide/1-hydroxybenzotriazole (DIC/HOBt) method. The final deprotection was carried out with an anhydrous hydrogen fluoride/m-cresol 10:1 at 0°C for 60 min. All the peptides have been purified using HPLC, and have purity above 95%. Peptide structures were confirmed by mass-spectrum analysis.

### Animals immunization

Spinal cord homogenate (SCH) was prepared from rats (non-linear specimen) as previously described (Stromnes and Goverman, 2006). Selected DA rats, each of 220–250 g in weight, were injected into hind footpads (100 μl/footpad) with spinal cord homogenate in incomplete Freund's adjuvant (1:1 w/v). Next day after immunization, the immunocortin started to be injected intraperitoneally (IP) to rats from experimental group (10 animals) on a daily basis. Daily dosage of the peptide was 400 μg/kg in a total volume of 100 μl per animal in 0.9% NaCl solution. The control group (10 animals) was given daily injections into hind footpads of 0.9% NaCl (100 μl). All rats in experiment were twice a day examined for clinical signs of EAE. Additionally, the body mass of animals were daily documented. Scores to grade clinical signs of the disease were taken as follow: 0—asymptomatic; 1—loss of tail tonicity; 2—impaired righting reflex; 3—partial paralysis; 4—complete paralysis; 5—moribund state or dead animals. Clinical sign with lower grading of the described symptoms was scored 0.5 points lower than the grade indicated. The mean EAE score ± SEM value was calculated. Typical EAE onset was observed between 10 and 11 days after immunization with maximum of the disease on 13 to 15 days. Clinical symptoms of the disease lasted for up-to 8–9 days.

### Lymph node lymphocyte culture

The lymph node lymphocytes (LN lymphocytes) were extracted from the lymph nodes of DA rats in 7 days after the animals immunization with the rat spinal cord homogenate as described (Stromnes and Goverman, 2006). Lymph nodes were collected aseptically from euthanized rats and single-cell suspensions were prepared by pressing the lymph node cells through a 40 μm mesh (Falcon™ Cell Strainer) with the help of a sterile syringe plunger. LN lymphocytes were isolated by Ficoll density gradient centrifugation (*d* = 1.077 g/ml, BioloT, St.Petersburg, Russia). LN lymphocytes were washed twice with Hank's Balanced Salt Solution (HBSS) and live cells were counted with Trypan Blue. Cell were propagated in DMEM medium supplemented with 10% fetal bovine serum, 2 mM glutamine, 2 mM sodium pyruvate, 20 mM 2-Mercaptoethanol and 40 μg/ml gentamicin. All media and chemicals were purchased from Sigma-Aldrich (USA).

### Radioactive isotopes

[^3^H]thymidine ([^3^H]TdR, 6,7 Ci/mmol) was purchased from Amersham Corp (UK).

### Cell proliferation assay

Proliferation assay of LN lymphocytes was performed in triplicate in 96-well plates. Cells (5 × 10^4^ per well) were incubated either with or without tested peptide (10 μg /ml) or 5 μg /ml ConA for 48 h. [^3^H]TdR (0.2 μCi) was added to each well and cells were incubated for 12 h. The cells were transferred to the GF/C filters (Whatman), washed twice with 10% trichloracetic acid followed by methanol. Incorporated radioactivity was counted in dpm using liquid scintillation (Beckman Instruments, Fullerton, CA).

### Statistical analysis

Data from EAE experiments were evaluated with Friedman's ANOVA and statistical analysis was performed using IBM SPSS Statistics v.22.

### Histology

Spinal cord sections were made from tissue taken for an analysis from immunocortin-injected animals, which were sacrificed on 15 day (the peak of the disease) after immunization with spinal cord homogenate. Spinal cord was fixed in ice-cold 10% methanol and embedded in paraffin before microtome sections (5 μm) were prepared to be stained with hematoxylin and eosin.

## Results

### Efficacy of the immunocortin *in vivo*

**Figure 1:**
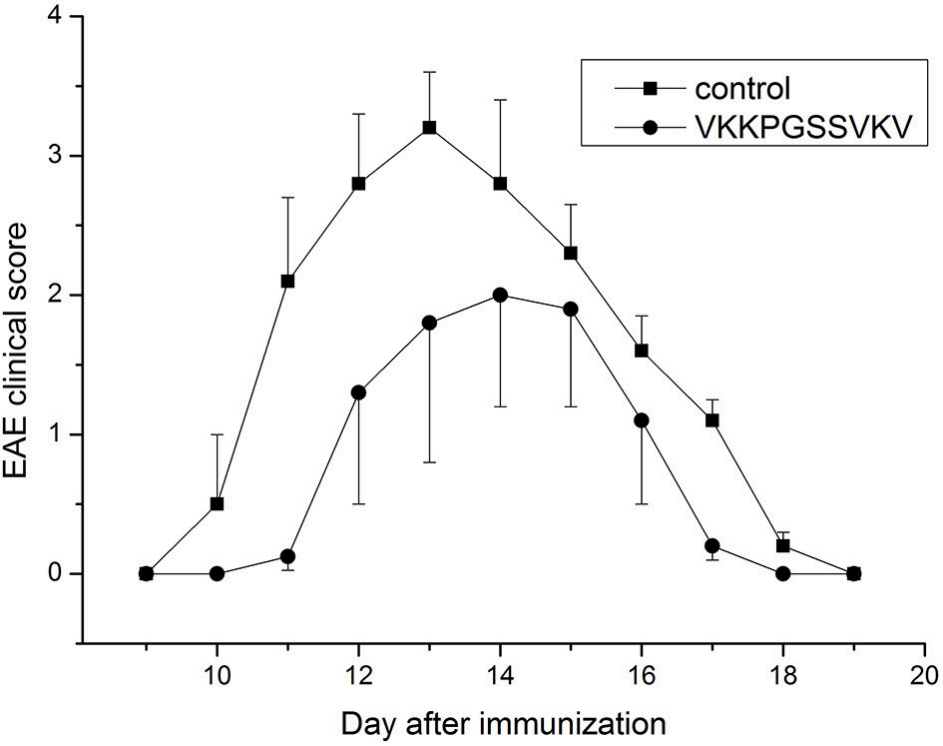
The expression of clinical signs of EAE in adult rats after immunocortin (VKKPGSSVKV) treatment. EAE clinical score was determined as described in Materials and methods. Rats were treated daily with immunocortin 400 μg/kg or normal saline (control). Each value shown is the mean ± SEM (*n* = 10) of clinical disease, which was scored from day 9 post-immunization (p.i.) until day 19 p.i. (*p* < 0.01, by Friedman's ANOVA). Calculated SEM are presented on the graph as error bars in the direction of plus or minus to avoid overlapping.

Some data on immunosuppressive activity of the immunocortin *in vitro* were published before (Mitin et al., 1993), and coincide with the idea of targeting the pathogenic lymphocytes generated in course of EAE development *in vivo* with the peptide. It was previously shown that intro-cerebroventricular injection (ICVI) of the immunocortin caused minor pyrogenic effect, and raised body temperature of rats by 0.7–1.3°C (Mitin et al., 1993). Evaluation of other systemic effects, which could result from the immunocortin usage required additional efforts. Since that time, we decided to investigate immunosuppressive activity of the immunocortin *in vivo* by focusing on the topic of whether it would improve the clinical symptoms in DA rats induced with EAE. By the study we were able to establish that intro-peritoneal injections of the immunocortin (400 μg/kg) clearly reduced severity of the EAE clinical symptoms in rats (Figure 1), and most importantly was that degree of clinical symptoms improvement was not accompanied by any sign of immunocortin toxicity to animals.

**Figure 2:**
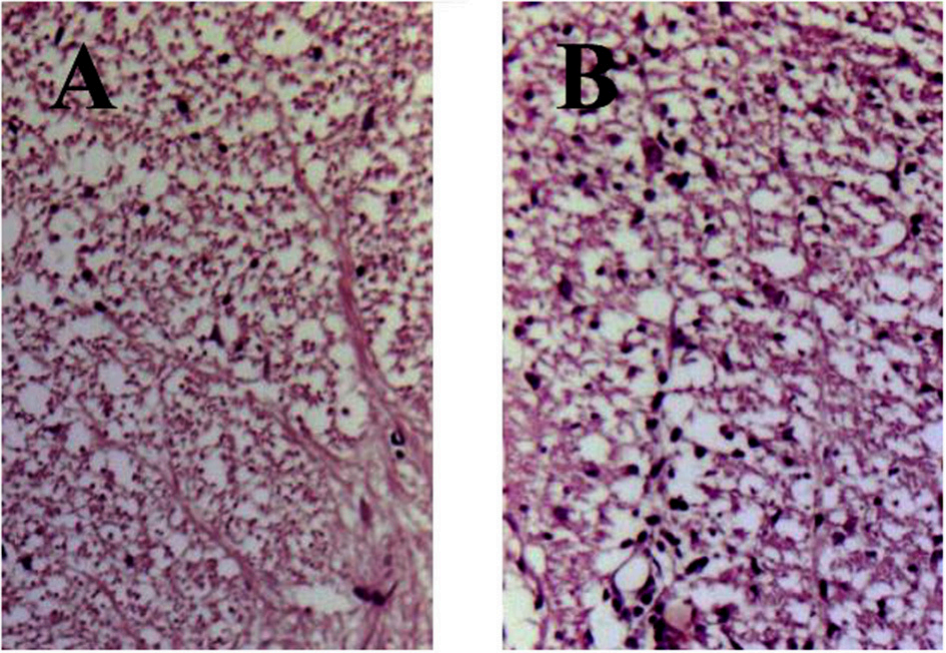
EAE rats histology of spinal cord sections stained with hematoxylin and eosin (day 13, p.i.). Rats were treated daily with immunocortin 400 μg/kg **(A)** or normal saline **(B)**.

Histology of eosin/hematoxylin stained sections showed less extensive perivascular and parenchymal signs of inflammation seen as of heavily stained local regions over the spinal cord volume in treated animals (Figure 2A). In contrast, spinal cord section taken from DA rats immunized with SCH but not treated with immunocortin has multiple signs of inflammation seen as numerous infiltrates (Figure 2B).

### Comparison analysis of MBP, and MBP P63–81 peptide effects on LN lymphocyte proliferation

**Figure 3:**
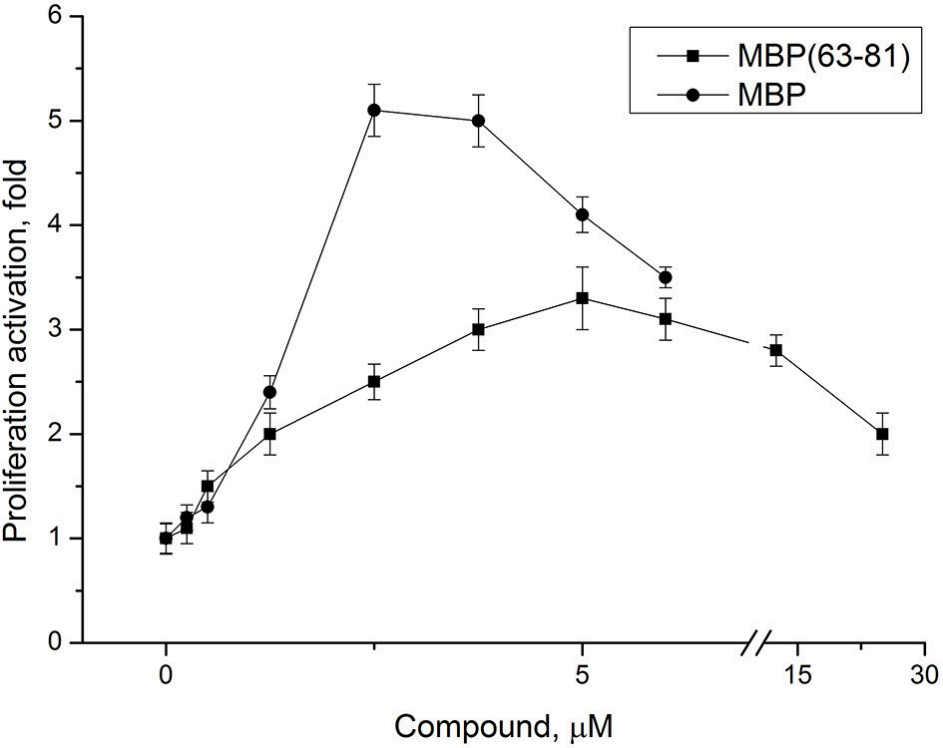
Dose-dependent effect of MBP(63-81) and MBP on LN lymphocyte proliferation. LN lymphocytes were isolated from lymph nodes of EAE rats. Each value shown is the mean ± SEM (*n* = 4). *p* < 0.05, by Friedman's ANOVA.

The peptide (63–81) of myelin basic protein (MBP) includes a common encephalitogenic immune dominant established for DA rats (Royland et al., 1992), yet its effective concentration has not been fully characterized for *in vitro* experiments. We studied dose-dependent effect of the peptide MBP(63–81) on proliferation of LN lymphocytes from EAE rats. MBP was purified as described previously (Navolotskaya et al., 2000), and used in experiments as a positive control. Both compounds effectively induce LN lymphocyte proliferation and MBP was more active in stimulating LN lymphocytes due to the presence on MBP of more than one antigenic determinant (Figure 3). Based on these data, the peptide optimal concentration for studying specific antiproliferative activity of the immunocortin *in vitro* was established.

### The immunocortin peptide inhibits antigen-dependent proliferation of LN lymphocytes from EAE rats in a dose-dependent manner

**Figure 4:**
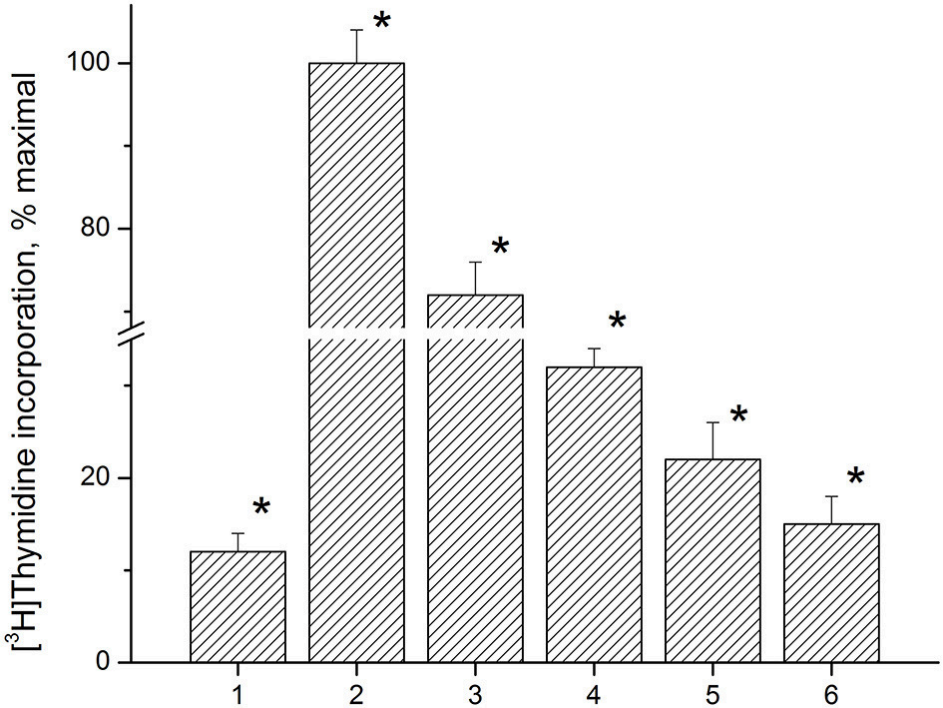
Analysis of anti-proliferative activity of immunocortin on LN lymphocytes from EAE rats stimulated with ConA and MBP(63–81). 1, negative control, non-stimulated LN lymphocytes; 2, proliferation of LN lymphocytes stimulated with ConA (5 μg/ml); 3, proliferation of LN lymphocytes stimulated with ConA (5 μg/ml) in the presence of immunocortin (10 μM); 4, proliferation of LN lymphocytes stimulated with 5 μM MBP(63–81); 5, proliferation of LN lymphocytes stimulated with 5 μM MBP(63–81) in the presence of immunocortin (1 μM); 6, proliferation of LN lymphocytes stimulated with 5 μM MBP(63–81) in the presence of immunocortin (10 μM). Each value shown is the mean ± SEM (*n* = 4).^*^ *p* < 0.05, by Friedman's ANOVA.

Anti-proliferative activity of the immunocortin was tested using LN lymphocytes from EAE rats. Con A (5 μg/ml) induced lymphocyte proliferation was used as a control. MBP(63–81) at 5 μM, and the immunocortin at 1 μM or 10 μM were added to the lymphocytes culture for 48 h. [^3^H]TdR labeled DNA precursor (0.2 μCi/well) was added and DNA- incorporated isotope quantity was measured by using a liquid scintillation counter (Figure 4). It was shown the immunocortin is able to inhibit MBP(63–81)-induced LN lymphocyte proliferation in a dose-dependent manner with maximum effect observed at the concentration 10 μM. The immunocortin could inhibit Con A-induced proliferation of LN lymphocytes from EAE rats. Indeed, the immunocortin inhibits LN lymphocyte proliferation induced with MBP (63–81), while data show that proliferation inhibiting effect of the immunocortin on LN lymphocytes can be established even with use of non-specific LN lymphocyte activator such as Con A.

## Discussion

Our data obtained *in vitro* and *in vivo* give an evidence immunosuppressive activity in the immunocortin. We for the first time demonstrated the immunocortin is effective in treatment of DA rats with EAE, which is a model of MS in humans. Administration of the immunocortin at daily dose up-to 400 μg/kg did not induce visible side effects in the experimental animals but efficiently suppressed specific EAE symptoms. Although the overall length of EAE course was not affected by treatment with the immunocortin, the disease symptoms severity was substantially lower in animals with EAE treated with the immunocortin.

We were able to show the immunocortin interferes with MBP(63–81) stimulated proliferation of LN lymphocytes collected from lymph nodes of EAE rats. At the same time, the immunocortin could inhibit proliferation of Con A stimulated LN lymphocytes, which proves the immunosuppressive effects of the peptide on MBP(63–81), and Con A stimulated LN lymphocytes have rather a common grounds. Our results partly support *in vitro* findings published earlier (Navolotskaya et al., 2000), when the immunocortin was shown *in vitro* to act immunosuppressive on mouse thymocytes, and also by reducing the bactericide activity, and spontaneous motility of peritoneal macrophages. Both thymocytes, and macrophages were shown to have high affinity receptor for ACTH-like peptide, and characteristics of specific binding kinetic for ACTH-like peptide, and ^125^I-labeled ACTH(13–24) peptide (ACTH “address segment”) were established. Among the effects of ACTH-like peptide on the target cells there were detected an increase in adenylate cyclase activity, and intracellular cAMP content. At the same time, there is a report that treatment with exogenous ACTH aggravated symptoms in MS patients with deficiency of endogenous ACTH (Matias-Guiu et al., 2011).

One may speculate the immunocortin based treatment could be effective for MS treatment. This assumption is based on the fact the immunocortin stimulates synthesis of endogenous sterol immunosuppressants by adrenal glands (e.g., cortisol) (Roelfsema et al., 2016), improve the local blood microcirculation in CNS (Van Bergen et al., 1996), and normalize the overall response to stress caused by the disease (Schäfer et al., 1997). Corticotropin releasing factors (CRFs) play a major role at the level of the hypothalamus, and pituitary glands to control stress-response mechanisms. Likely to CRF, endogenous immunocortin is mostly produced outside CNS. Therefore it could induce some paracrine mechanisms. ACTH receptor could be a potential target for the immunocortin. One can speculate that IVIG therapy may accelerate production of endogenous immunocortin due to enhanced degradation of immunoglobulins in macrophages and monocytes. This effect may contribute to overall IVIG therapy anti-MS efficacy beyond the hypothesized displacement of anti-myelin antibodies from the blood circulation with normal donor immunoglobulins.

Details of that peptide activity affecting many tissues remain unresolved even the immunocortin was shown to influence the immune cells migration, and imflammasomes degradation (Barclay and Shinohara, 2016; Bulatov et al., 2016). The peptide might induce opioid peptides release from the immune cells thus inhibiting pain stimuli transmission to peripheral sensory nerve receptors. Peripheral paracrine effects of the immunocortin may be similar to hypothalamic ACTH action, which counterbalances some stress stimuli, such as local inflammation and pain (Khaibullin et al., 2017).

Considering relatively low effective concentration of the immunocortin registered *in vitro* (15–20% inhibition of immune cell proliferation at 10 mM peptide concentration), one should take in account significant difference of Ig VH1 domain sequences in human and rat. Indeed, one amino acid residue of the immunocortin (Val10) does not match corresponding position in rat Ig heavy chain. Substitution of Val10 → Ile in the immunocortin structure should make it closer to rat Ig, and thus elevate its immunosuppressive efficiency in rat, while proportionally decreasing its activity in humans.

Taken together, our data allow considering the immunocortin as a candidate agent for treatment of MS. Similarly to other drugs used for MS treatment, the immunocortin does not affect antigen-dependent mechanisms of the disease. Therefore, it cannot be used as a pathogenesis specific therapeutic. However, endogenous origin of the immunocortin allows expecting less pronounced undesirable side effects than in synthetic immunosuppressants.

## Author contributions

VT and AD: Conception and design of study, Peptide structure analysis, Acquisition of data, Data analysis and interpretation. AS: Conception and design of study, Data analysis and interpretation, Drafting the article. YB: Acquisition of data, Drafting the article. NP and VA: Acquisition of data, Data analysis and interpretation. AM: Conception and design of study, Critical revision of the article. VL: Data analysis and interpretation, Critical revision of the article. IU: Conception and design of study, Data analysis and interpretation, Drafting the article, Critical revision of the article.

### Conflict of interest statement

The authors declare that the research was conducted in the absence of any commercial or financial relationships that could be construed as a potential conflict of interest.
